# 

**Published:** 1903-08

**Authors:** 


SUBSCRIPTION $1.00.

PUBLISHED MONTHLY

### ATLANTA JOURNAL-RECORD OF MEDICINE.

Successor to Atlanta Medical and Surgical Journal, Established 1855, and Southern Medical Record, Established 1870.

Vol. V. | Atlanta, Ga., August, 1903. | No. 5.

